# Higher serum phosphorus and calcium levels provide prognostic value in patients with acute myocardial infarction

**DOI:** 10.3389/fcvm.2022.929634

**Published:** 2022-09-07

**Authors:** Wei Cao, Yilan Li, Yao Wen, Shaohong Fang, Bing Zhao, Xiaoyuan Zhang, Yanxiu Zhang, Xueyan Lang, Bo Yu, Yao Zhang

**Affiliations:** ^1^Department of Cardiology, Second Affiliated Hospital of Harbin Medical University, Harbin, China; ^2^Department of Cardiology, Heilongjiang Provincial Hospital, Harbin, China; ^3^Myocardial Ischemia, Ministry of Education, Harbin Medical University, Harbin, China

**Keywords:** serum phosphate, serum calcium, acute myocardial infarction, prognosis, heart failure, mortality

## Abstract

**Background:**

Although traditional cardiovascular risk factors are closely related to the poor prognosis of acute myocardial infarction (AMI) patients, there are few studies on the relationship of serum phosphorus and calcium with prognosis in AMI patients. The relationship of serum phosphorus and calcium with prognostic biomarkers in AMI remains unclear.

**Methods and results:**

A total of 3,891 AMI patients were enrolled from a prospective cohort study. We investigated the association of serum phosphorus and calcium with prognostic biomarkers. The risk of in-hospital heart failure (HF), post-discharge HF, all-cause mortality and cardiac mortality was estimated across quartiles of serum phosphorus and calcium levels. Serum phosphorus and calcium levels were associated with biomarkers of prognosis. Overall, 969 patients developed in-hospital HF during hospitalization, 549 patients developed post-discharge HF during a median follow-up of 12 months, and 252 patients died, with 170 cardiac deaths since admission. In the fully adjusted model, compared with patients in quartile 2 (Q2), patients with serum phosphorus levels in Q4 were at greater risk of post-discharge HF [sub-distributional hazard ratios (SHR) 1.55; 95% confidence interval (CI), 1.21–1.99], in-hospital HF [odds ratio (OR) 1.84; 95% CI, 1.47–2.31], all-cause mortality (HR 1.59; 95% CI, 1.08–2.32), and cardiac mortality (SHR 1.68; 95% CI, 1.03–2.75). Compared with patients in Q2, patients with corrected calcium levels in Q4 had a higher risk of in-hospital HF (OR 1.62; 95% CI, 1.29–2.04), all-cause mortality (HR 1.99; 95% CI, 1.37–2.88), and cardiac mortality (SHR 1.87; 95% CI, 1.19–2.96; all *p*-trend < 0.05).

**Conclusion:**

Serum phosphorus and calcium levels were associated with AMI prognostic biomarkers in AMI. Higher serum phosphorus was independently related to the increased risk of in-hospital HF, postdischarge HF, all-cause mortality and cardiac mortality, and higher serum calcium was independently related to the increased risk of in-hospital HF, all-cause mortality and cardiac mortality after AMI.

## Introduction

Patients with acute myocardial infarction (AMI) have a high risk of heart failure (HF) and mortality (1). Although advances in the diagnosis and treatment of AMI have reduced the risk of mortality in AMI patients, the incidence of HF is still on the rise after myocardial infarction (MI), which significantly increases the risk of short-term and long-term adverse events in AMI patients (2). Although some prognostic biomarkers (such as troponin, natriuretic peptide, and C-reactive protein levels) are related to poor prognosis after MI (3–5), there are still great challenges in the early prediction of clinical outcomes.

In the past, more attention has been given to the correlation between poor prognosis and traditional cardiovascular risk factors such as blood glucose, blood lipids, and smoking in AMI patients. However, prior studies have shown that phosphorus and calcium are associated with cardiovascular disease (CVD) as non-traditional risk factors (6–11).

Although most studies found that higher serum phosphate levels were related to post-discharge HF risk or mortality in various populations, including MI (6–9), controversy remained in another study (12), in which there was no relationship between serum phosphorus and cardiovascular events.

In addition, the link between serum calcium levels and poor outcome has been debated in different populations. Lower serum calcium levels were related to the risk of all-cause death in patients with acute coronary syndrome (ACS) (10) or in HF with preserved EF (HFpEF) patients (13), but both high and low serum calcium levels were related to an increased risk of in-hospital mortality in another study (14). Moreover, higher serum calcium levels were associated with HF risk in a community-based cohort study (11).

Prior studies were carried out in a part of the MI population or non-MI population alone, and most were limited by small sample sizes. Few studies have evaluated the relationship between serum phosphorus and serum calcium levels with in-hospital HF. In this study, we systematically evaluated the prognostic value of serum phosphorus and serum calcium levels on in-hospital HF, post-discharge HF, all-cause mortality, and cardiac mortality in a large AMI population.

## Methods

### Study design and population

**Figure 1:**
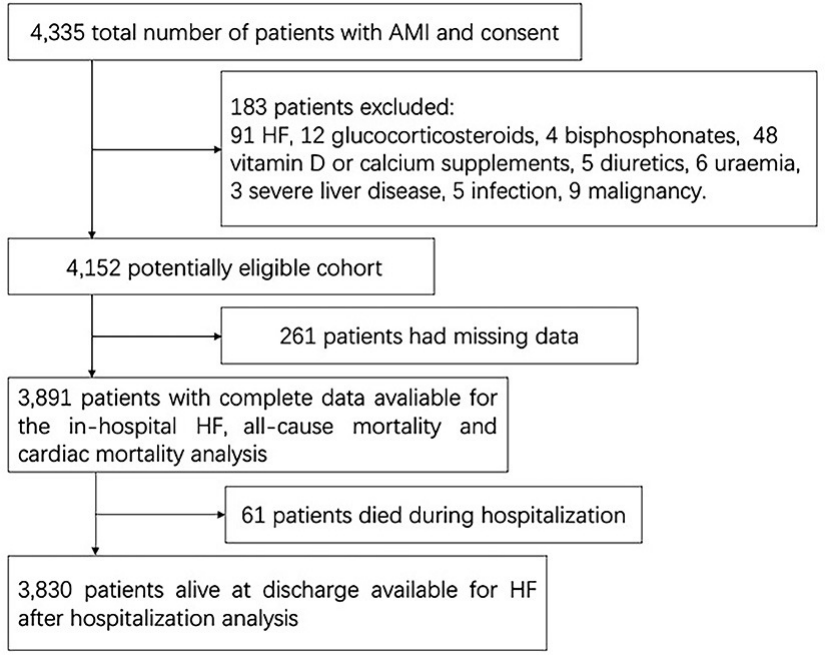
Study sample. AMI, acute myocardial infarction; HF, heart failure.

All patients were from a prospective hospital-based AMI cohort study, which was supported by the National Key Research and Development Program of China. From 1 April 2017 to 31 March 2019, 4,335 patients with AMI, aged ≥18 years, were admitted to the Department of Cardiology of the Second Affiliated Hospital of Harbin Medical University. Patients were excluded if they met any of the following criteria: (1) treatment with glucocorticosteroids, bisphosphonates, calcium or vitamin D supplements, or diuretics within the past 1 month; (2) medical history of HF, active infection, liver disease, uremia [estimated glomerular filtration rate (eGFR) lower than 15 mL/min/1.73 m^2^, or dialysis or transplantation is needed], or malignancy; and (3) insufficient medical records (Figure 1). A total of 3,891 patients were finally included in this complete data cohort. A total of 3,830 patients who survived at discharge agreed to be followed.

All patients were followed from admission to death or the last follow-up date. The regular follow-up times were 1, 3, 6, 12, 18, and 24 months.

### Definitions

AMI should be diagnosed according to the Fourth Universal Definition of Myocardial Infarction (2018) (15). HF after AMI was defined as typical signs and symptoms of congestion that required treatment with a diuretic or intravenous vasodilator. Participants with N-terminal pro-B-type natriuretic peptide (NT-proBNP) < 125 pg/mL in the non-acute setting or NT-proBNP < 300 pg/mL in the acute setting were excluded from HF (16). Multi-vessel disease was defined as stenosis of at least two main blood vessels (≥2 mm diameter) >70%, measured by quantitative coronary angiography (17).

### Common clinical and laboratory assessments

We collected blood samples on admission and sent them to the laboratory for testing immediately. Serum phosphorus was measured by using methods based on ammonium molybdate, and serum calcium was measured with an approach based on o-cresolphthalein complexone on the Siemens Dimension Clinical Chemistry System. The baseline population characteristics were collected from medical records, prior medication, and self-reports. Echocardiography characteristics were examined at admission and reviewed when necessary.

### Equations

The serum calcium level was corrected according to the formula: albumin-corrected serum calcium (mmol/L) = measured serum calcium (mmol/L) – 0.025^*^ serum albumin (g/L) +1.0 (mmol/L) (18).

### Clinical outcomes

The primary outcome was post-discharge HF after AMI. The secondary outcomes were in-hospital HF and all-cause death, including cardiac death after AMI. Cardiac death was defined as death due to recurrent MI (RMI), HF, severe arrhythmias and sudden death (19).

### Statistics analysis

For baseline characteristics, the median [interquartile range (IQR)] or number (percentage) was used as appropriate. Serum phosphorus and calcium levels were analyzed as continuous variables and as quartiles. Characteristics among different serum phosphorus and corrected serum calcium quartile groups were compare by Kruskal–Wallis test, chi-square test or Fisher's exact test. We used multivariate multiple imputation with chained equations to impute missing values. The correlation of serum phosphorus and calcium with prognostic biomarkers was assessed by Spearman correlation analysis. The non-linear association of serum phosphorus and calcium levels with clinical outcomes was examined by restricted cubic splines. Kaplan–Meier curves were used to perform the proportion of patients free of clinical outcomes according to quartiles of serum phosphorus or corrected serum calcium, which were compared by log-rank. The association of serum phosphorus or calcium with the incidence of in-hospital HF was evaluated by logistic regression analysis. Cox proportional hazards models were used to calculate hazard ratios (HRs) and 95% CIs for all-cause mortality. Fine-Gray proportional sub-distribution hazards models were used to evaluate the relationship of serum phosphorus and calcium levels with the incidence of post-discharge HF or cardiac death, and death without HF was regarded as a competitive risk of post-discharge HF and non-cardiac death as a competitive risk of cardiac death. The regression results were assessed according to serum phosphorus or corrected serum calcium quartiles using quartile 2 (Q2) as the reference. Four multivariable models were constructed. The first model was adjusted according to sex, age, and body mass index (BMI). In Model 2, AMI types, smoking status, prior history of hypertension, diabetes, MI, percutaneous coronary intervention (PCI) and coronary artery bypass grafting (CABG) were further adjusted. In Model 3, heart rate, PCI information and multi-vessel disease were further adjusted. In Model 4, cardiac troponin I (cTNI), NT-proBNP, eGFR, total cholesterol (TC), triglyceride (TG) two kinds of serum phosphorus, corrected serum calcium and magnesium were further adjusted. Stratified analysis based on subgroup variables was performed with full adjustments. Interaction terms were used to evaluate whether subgroup variables modified the associations between serum phosphorus and calcium with the relative risk of clinical outcomes. Stata Statistical Software (Version 15.1; Stata Corp, College Station, TX, USA), and IBM SPSS Statistics (Version 25.0; IBM, Armonk, NY, USA) were used for all statistical analyses, and the results were considered statistically significant at *P* < 0.05.

## Results

### Baseline characteristics

Among the 3,891 participants, 67.9% were ST-segment elevation MI (STEMI) patients, 69.1% were male, and the median age was 61 years (IQR 53–69). The median serum phosphate level was 1.21 mmol/L (IQR 1.07–1.36), and the median corrected serum calcium level was 2.17 mmol/L (IQR 2.09–2.27) on hospital admission. According to quartiles of serum phosphate and corrected serum calcium, the cohort characteristics are shown in Tables 1, 2.

**Table 1:** Participant characteristics by quartiles of serum phosphorus at baseline.

**Patients with complete data on all baseline variables**						
	**Overall** ***n*** **= 3,891**	**Serum phosphorus Q1, mmol/L**	**Serum phosphorus Q2, mmol/L**	**Serum phosphorus Q3, mmol/L**	**Serum phosphorus Q4, mmol/L**	
		**≤ 1.07**	**1.08–1.21**	**1.21–1.36**	**≥1.36**	***P*** **-value**
		***n*** **= 1,025**	***n*** **= 953**	***n*** **= 991**	***n*** **= 922**	
Age (years)	61 (53–69)	63 (55–70)	61 (53–68)	60 (50–68)	61 (52–68)	<0.001
Male	2,688 (69.1)	829 (80.9)	711 (74.6)	657 (66.3)	491 (53.3)	<0.001
BMI (kg/m^2^)	24.7 (22.5–27.4)	24.8 (22.8–27.4)	24.8 (22.5–27.4)	24.8 (22.5–27.4)	24.5 (22.3–27.3)	0.237
SBP (mmHg)	132 (117–150)	135 (120–150)	131 (119–149)	133 (116–150)	131 (113–150)	0.018
Heart rate (beats/min)	81 (71–90)	82 (72–90)	81 (73–91)	81 (70–90)	80 (70–90)	0.194
**Previous history**						
Hypertension	2,030 (52.2)	526 (51.3)	486 (51.0)	499 (50.4)	519 (56.3)	0.038
Diabetes	909 (23.4)	212(20.7)	195 (20.5)	244 (24.6)	258 (28.0)	<0.001
MI	381 (9.8)	113 (11.0)	97 (10.2)	90 (9.1)	81 (8.8)	0.315
Prior PCI	248 (6.4)	78 (7.6)	60 (6.3)	53 (5.3)	57 (6.2)	0.218
Prior CABG	4 (0.1)	0 (0)	2 (0.2)	2 (0.2)	0 (0)	0.26
Smoking (current+ex)	2,533 (65.1)	663 (64.7)	644 (67.6)	667 (67.3)	559 (60.6)	0.005
**AMI types**						
STEMI	2,642 (67.9)	676 (66.0)	646 (67.8)	691 (69.7)	629 (68.2)	0.34
CAG	3,757 (96.6)	993 (96.9)	923 (96.9)	954 (96.3)	887 (96.2)	0.762
PCI	3,243 (83.3)	837 (81.7)	810 (85.0)	814 (82.1)	782 (84.8)	0.092
Multi-vessel disease	1,701 (43.7)	416 (40.6)	418 (43.9)	422 (42.6)	445 (48.3)	0.006
**Echocardiographic characteristics**						
LAD (mm)	34.8 (32.0–37.9)	35.0 (32.4–37.9)	34.7 (32.0–37.9)	34.7 (31.9–37.7)	34.8 (31.7–38.0)	0.260
LVEDD (mm)	46.0 (42.9–49.0)	46.0 (43.1–48.9)	46.0 (42.6–49.1)	45.8 (42.7–49.0)	46.0 (42.9–49.4)	0.611
IVSD (mm)	10.6 (9.8–11.7)	10.6 (9.8–11.7)	10.5 (9.8–11.6)	10.6 (9.7–11.7)	10.6 (9.7–11.6)	0.275
EF (%)	61.0 (54.0–62.0)	61.0 (55.0–62.0)	61.0 (55.0–62.0)	61.0 (54.0–62.0)	59.0 (50.0–62.0)	<0.001
**Laboratory covariates**						
cTNI (ng/L)	31.6 (8.5–94.9)	27.5 (7.7–81.2)	32.0 (8.0–98.3)	31.5 (8.3–92.9)	37.4 (10.9–109.7)	0.002
NT–proBNP (pmol/L)	1,003.0 (343.0–2,630.0)	808.0 (267.0–1,971.5)	928.0 (315.5–2,177.0)	910.0 (328.0–2,637.0)	1,571.5 (548.8–4,590.8)	<0.001
hs–CRP (mg/L)	5.7 (2.3–11.8)	5.1 (2.1–12.3)	5.2 (2.2–11.7)	5.5 (2.4–11.4)	7.3 (2.8–12.1)	<0.001
eGFR (mL/min/1.73 m^2^)	82.3 (66.1–95.0)	81.5 (68.2–93.3)	83.7 (67.6–96.0)	83.6 (67.5–96.3)	79.6 (58.3–94.6)	<0.001
Serum calcium (mmol/L)	2.22 (2.13–2.30)	2.21 (2.13–2.29)	2.21 (2.13–2.30)	2.22 (2.14–2.31)	2.22 (2.12–2.32)	0.090
Corrected calcium (mmol/L)	2.17 (2.09–2.27)	2.17 (2.10–2.27)	2.16 (2.08–2.25)	2.17 (2.09–2.26)	2.18 (2.10–2.28)	0.004
Serum magnesium (mmol/L)	0.85 (0.79–0.92)	0.85 (0.78–0.92)	0.85 (0.79–0.92)	0.85 (0.79–0.92)	0.86 (0.79–0.92)	0.218
Serum albumin (g/L)	41.6 (39.0–44.2)	41.3 (39.0–43.7)	42.0 (39.5–44.4)	42.1 (39.6–44.7)	41.2 (38.1–44.2)	<0.001
TG (mmol/L)	1.38 (0.98–1.98)	1.25 (0.92–1.81)	1.34 (0.94–1.92)	1.50 (1.01–2.11)	1.49 (1.03–2.15)	<0.001
TC (mmol/L)	4.55 (3.90–5.27)	4.44 (3.80–5.05)	4.52 (3.86–5.22)	4.68 (3.98–5.46)	4.62 (3.94–5.44)	<0.001
**Medical treatment**						
Aspirin	3,779 (97.1)	1,001 (97.7)	930 (97.6)	963 (97.2)	885 (96.0)	0.109
Clopidogrel or ticagrelor	3,797 (97.6)	1,004 (98.0)	933 (97.9)	972 (98.1)	888 (96.3)	0.039
Statins	3,755 (96.5)	1,001 (97.7)	923 (96.9)	958 (96.7)	873 (94.7)	0.004
β Blocker	3,743 (96.2)	984 (96.0)	925 (97.1)	959 (96.8)	875 (94.9)	0.067
ACEI/ARB	2,908 (74.7)	738 (72.0)	710 (74.5)	766 (77.3)	694 (75.3)	0.053

Values are median (25th, 75th percentile) or number (percentages).

ACEI, angiotensin-converting enzyme inhibitor; AMI, acute myocardial infarction; ARB, angiotensin II receptor blocker; BMI, body mass index; CABG, coronary artery bypass grafting; CAG, coronary angiography; cTNI, cardiac troponin I; EF, ejection fraction; eGFR, estimated glomerular filtration rate; hs-CRP, high-sensitivity C reactive protein; IVSD, interventricular septum, diastolic; LAD, left atrial diameter; LVEDD, left ventricular end-diastolic dimension; MI, myocardial infarction; NT-proBNP, N-terminal pro-B-type natriuretic peptide; PCI, percutaneous coronary intervention; Q, quartile; SBP, systolic blood pressure; STEMI, ST-segment elevation myocardial infarction; TC, total cholesterol; TG, triglyceride.

**Table 2:** Participant characteristics by quartiles of corrected serum calcium at baseline.

**Patients with complete data on all baseline variables**						
	**Overall**	**Corrected calcium Q1, mmol/L**	**Corrected calcium Q2, mmol/L**	**Corrected calcium Q3, mmol/L**	**Corrected calcium Q4, mmol/L**	
		** ≤ 2.09**	**2.09–2.17**	**2.17–2.27**	**≥2.27**	***P*** **-value**
	***n*** **= 3,891**	***n*** **= 977**	***n*** **= 979**	***n*** **= 974**	***n*** **= 961**	
Age (years)	61 (53–69)	59 (49–66)	60 (52–67)	62 (54–69)	64 (57–72)	<0.001
Male	2,688 (69.1)	751 (76.9)	725 (74.1)	647 (66.4)	565 (58.8)	<0.001
BMI (kg/m^2^)	24.7 (22.5–27.4)	25.3 (22.9–27.7)	24.8 (22.9–27.4)	24.6 (22.3–27.3)	24.3 (22.0–27.0)	<0.001
SBP (mmHg)	132 (117–150)	135 (117–150)	134 (120–150)	130 (116–150)	131 (115–150)	0.008
Heart rate (beats/min)	81 (71–90)	82 (73–93)	82 (74–90)	80 (71–90)	80 (70–90)	<0.001
**Previous history**						
Hypertension	2,030 (52.2)	493 (50.5)	503 (51.4)	514 (52.8)	520 (54.1)	0.395
Diabetes	909 (23.4)	197 (20.2)	200 (20.4)	229 (23.5)	283 (29.4)	<0.001
MI	381 (9.8)	98 (10.0)	92 (9.4)	97 (9.9)	94 (9.8)	0.966
Prior PCI	248 (6.4)	61 (6.0)	65 (6.8)	64 (6.5)	58 (5.9)	0.942
Prior CABG	4 (0.4)	1 (0.1)	0	0	3 (0.3)	0.046
Smoking (current+ex)	2,533 (65.1)	684 (70.0)	655 (66.9)	611 (62.7)	583 (60.7)	<0.001
**AMI types**						
STEMI	2,642 (67.9)	711 (72.8)	668 (68.2)	649 (66.6)	614 (63.9)	<0.001
CAG	3,757 (96.6)	956 (97.9)	949 (96.9)	944 (96.9)	908 (94.5)	<0.001
PCI	3,243 (83.3)	831 (85.1)	819 (83.7)	822 (84.4)	771 (80.2)	0.022
Multivessel disease	1,701 (43.7)	408 (41.8)	436 (44.5)	416 (42.7)	441 (45.9)	0.258
**Echocardiographic characteristics**						
LAD (mm)	34.8 (32.0–37.9)	34.4 (31.9–37.7)	34.5 (31.9–37.5)	34.9 (32.0–38.0)	35.4 (32.4–38.2)	0.113
LVEDD (mm)	46.0 (42.9–49.0)	45.8 (43.0–48.7)	45.9 (43.0–48.7)	46.0 (43.0–49.3)	46.1 (42.4–50.0)	0.360
IVSD (mm)	10.6 (9.8–11.7)	10.6 (9.7–11.7)	10.7 (9.9–11.7)	10.5 (9.8–11.6)	10.5 (9.7–11.6)	0.384
EF (%)	61.0 (54.0–62.0)	61.0 (55.0–63.0)	61.0 (55.0–62.0)	60.2 (54.0–62.0)	59.0 (51.0–62.0)	<0.001
**Laboratory covariates**						
cTNI (ng/L)	31.6 (8.5–94.9)	46.2 (12.8–113.7)	31.2 (8.2–94.4)	33.0 (9.3–95.8)	22.7 (6.6–75.2)	<0.001
NT–proBNP (pmol/L)	1,003.0 (343.0–2,630.0)	718.0 (256.5–1,742.0)	776.0 (271.0–1,862.0)	1,080.5 (379.8–2,631.3)	1,788.0 (614.5–4,504.5)	<0.001
hs–CRP (mg/L)	5.7 (2.3–11.8)	4.2 (1.8–10.6)	4.8 (2.0–10.9)	6.0 (2.4–12.1)	9.4 (3.4–13.5)	<0.001
eGFR (mL/min/1.73m^2^)	82.3 (66.1–95.0)	86.6 (72.6–97.8)	86.3 (70.4–97.6)	80.2 (64.8–94.2)	74.3 (58.3–89.0)	<0.001
Serum calcium (mmol/L)	2.22 (2.13–2.30)	2.10 (2.02–2.17)	2.19 (2.14–2.24)	2.25 (2.19–2.31)	2.32 (2.26–2.39)	<0.001
Serum magnesium (mmol/L)	0.85 (0.79–0.92)	0.85 (0.78–0.92)	0.86 (0.80–0.92)	0.85 (0.79–0.92)	0.85 (0.79–0.93)	0.766
Serum albumin (g/L)	41.6 (39.0–44.2)	43.5 (40.9–46.0)	42.4 (40.3–44.5)	41.2 (38.9–43.7)	39.4 (36.6–41.8)	<0.001
Serum phosphorus (mmol/L)	1.20 (1.07–1.35)	1.19 (1.08–1.33)	1.19 (1.05–1.33)	1.21 (1.06–1.35)	1.21 (1.07–1.38)	0.030
TG (mmol/L)	1.38 (0.98–1.98)	1.39 (0.97–2.05)	1.41 (0.99–2.00)	1.37 (0.97–1.98)	1.36 (0.98–1.94)	0.676
TC (mmol/L)	4.55 (3.90–5.27)	4.51 (3.94–5.27)	4.57 (3.90–5.24)	4.58 (3.92–5.37)	4.54 (3.81–5.26)	0.378
**Medical treatment**						
Aspirin	3,779 (97.1)	952 (97.4)	958 (97.9)	954 (97.9)	915 (95.2)	0.001
Clopidogrel or ticagrelor	3,797 (97.6)	956 (97.9)	962 (98.3)	955 (98.0)	924 (96.1)	0.009
Statins	3,755 (96.5)	942 (96.4)	953 (97.3)	945 (97.0)	915 (95.2)	0.055
β Blocker	3,743 (96.2)	951 (97.3)	952 (97.2)	936 (96.1)	904 (94.1)	<0.001
ACEI/ARB	2,908 (74.7)	766 (78.4)	744 (76.0)	706 (72.5)	692 (72.0)	0.003

Values are median (25th, 75th percentile) or number (percentages).

ACEI, angiotensin-converting enzyme inhibitor; AMI, acute myocardial infarction; ARB, angiotensin II receptor blocker; BMI, body mass index; CABG, coronary artery bypass grafting; CAG, coronary angiography; cTNI, cardiac troponin I; EF, ejection fraction; eGFR, estimated glomerular filtration rate; hs-CRP, high-sensitivity C reactive protein; IVSD, interventricular septum, diastolic; LAD, left atrial diameter; LVEDD, left ventricular end-diastolic dimension; MI, myocardial infarction; NT-proBNP, N-terminal pro-B-type natriuretic peptide; PCI, percutaneous coronary intervention; Q, quartile; SBP, systolic blood pressure; STEMI, ST-segment elevation myocardial infarction; TC, total cholesterol; TG, triglyceride.

Patients with higher phosphorus were more likely to be younger, females, and non-smokers and had a higher prevalence of hypertension, diabetes and multi-vessel diseases, lower systolic blood pressure, higher cTNI, higher NT-proBNP, higher hs-CRP, higher corrected serum calcium, higher TC and TG, lower EF, and lower eGFR. Patients with higher serum phosphorus were less likely to be treated with antiplatelet drugs and statins during hospitalization.

What's more, patients with higher corrected calcium were more likely to be older, females, non-smokers, and non-STEMI and had a higher prevalence of diabetes or prior CABG, lower BMI, lower systolic blood pressure, lower heart rate, lower cTNI, higher NT-proBNP, higher hs-CRP, higher serum phosphate, higher left atrial diameter, lower EF, lower eGFR and lower serum albumin. Patients with higher serum phosphorus were less likely to be treated with antiplatelet drugs, β- blockers, angiotensin converting enzyme inhibitors or angiotensin II receptor blockers, PCI, and coronary angiography during hospitalization.

### Correlation of phosphorus and calcium levels with prognostic biomarkers and echocardiographic parameters

With Spearman correlation analysis, serum phosphorus was positively correlated with NT-proBNP (*r* = 0.171; *p* < 0.001), hs-CRP (*r* = 0.066; *p* < 0.001), and cTNI (*r* = 0.058; *p* < 0.001) and negatively correlated with EF (*r* = −0.094; *p* < 0.001) and eGFR (*r* = −0.044; *p* = 0.006). Corrected serum calcium was also positively correlated with NT-proBNP (*r* = 0.230; *p* < 0.001) and hs-CRP (*r* = 0.207; *p* < 0.001) and negatively correlated with cTNI (*r* = −0.110; *p* < 0.001), EF (*r* = −0.095; *p* < 0.001) and eGFR (*r* = −0.225; *p* < 0.001; Supplementary Table S1).

### Clinical outcomes

The median follow-up was 12 months (range 12–24 months). During hospitalization, in-hospital HF occurred in 969 (24.9%) patients. During follow-up, 549 (14.3%) patients developed post-discharge HF. A total of 252 (6.6%) patients died, and 170 (4.4%) patients experienced cardiac death after AMI.

#### Serum phosphorus and post-discharge HF

**Figure 2:**
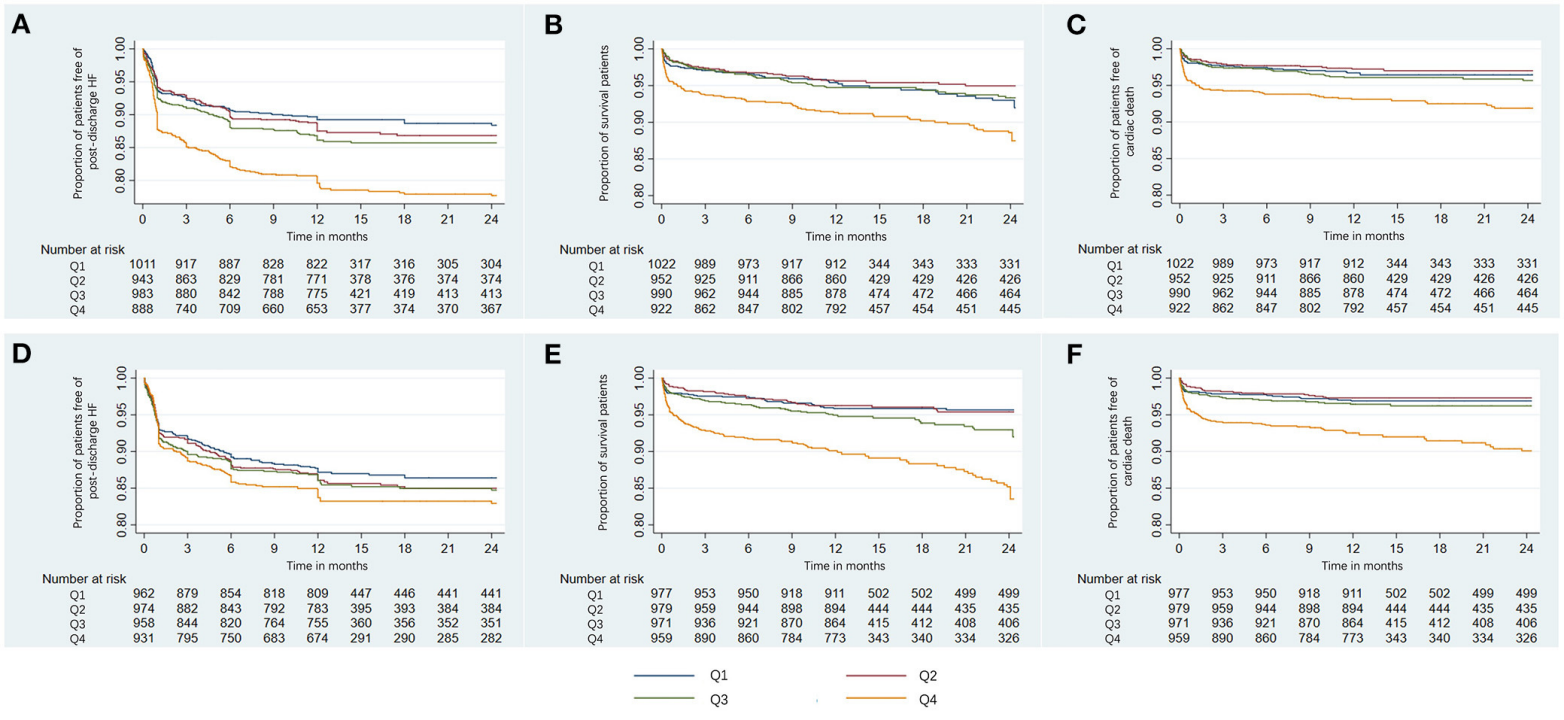
Kaplan-Meier curves of the proportion of AMI patients free of clinical outcomes. Shown are the proportion of patients free of post-discharge HF **(A)**, survival rate **(B)**, and the proportion of patients free of cardiac death **(C)** for serum phosphorus, and the proportion of patients free of post-discharge HF **(D)**, survival rate **(E)**, and the proportion of patients free of cardiac death **(F)** for corrected serum calcium. AMI, acute myocardial infarction; HF, heart failure; Q, quartile.

**Figure 3:**
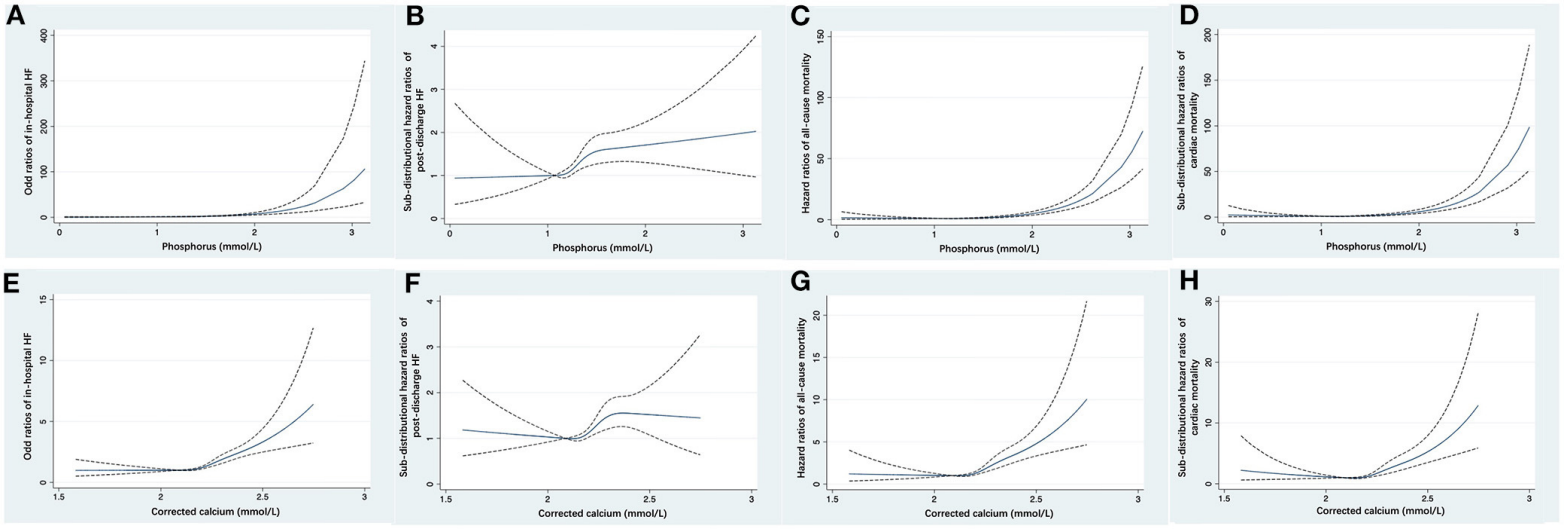
Restricted cubic spline fitting for the association of serum phosphorus with the risk of in-hospital HF **(A)**, post-discharge HF **(B)**, all-cause mortality **(C)**, and cardiac mortality **(D)**, and the association of corrected serum calcium with the risk of in-hospital HF **(E)**, post-discharge HF **(F)**, all-cause mortality **(G)**, and cardiac mortality **(H)**. ORs or HRs were evaluated based on a univariate logistic regression or Cox proportional regression model. The area between dotted lines represents the 95% CI. CI, confidence interval; HF, heart failure; HR, hazard ratio; OR, odds ratio.

With increasing quartiles of phosphorus levels, the proportion of patients free of post-discharge HF decreased at the 12-month median follow-up (Figure 2A). Serum phosphorus levels had a S-shaped relationship with post-discharge HF (in unadjusted analysis, Figure 3B).

**Figure 4:**
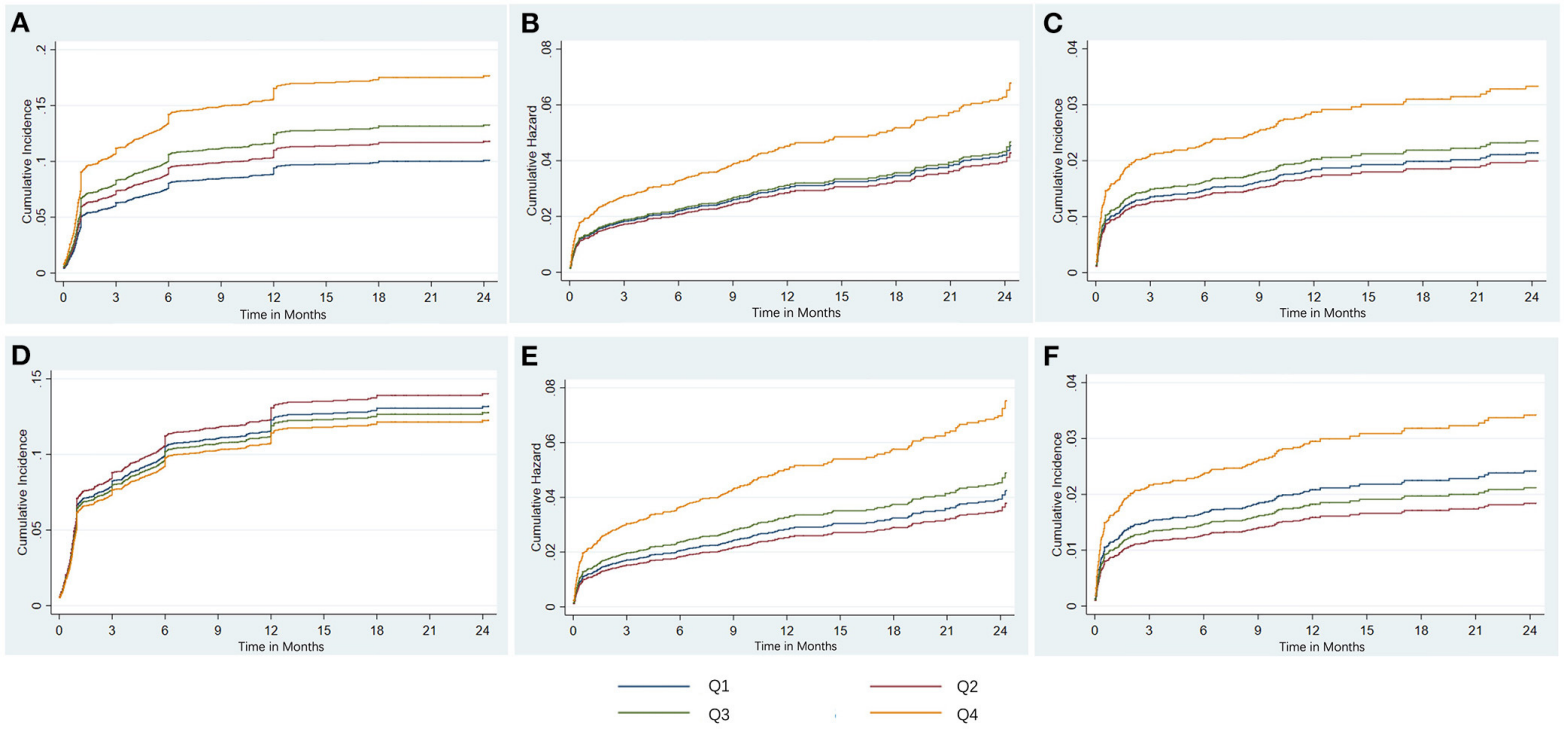
The risk regression analysis of clinical outcomes based on fully adjusted models. Shown are the cumulative incidence of post discharge HF treating death without HF as competing risk **(A)**, the cumulative hazard of all-cause death **(B)**, and the cumulative incidence of cardiac death treating non-cardiac death as competing risk **(C)** for serum phosphate, and the cumulative incidence of post-discharge HF treating death without HF as competing risk **(D)**, the cumulative hazard of all-cause death **(E)**, and the cumulative incidence of cardiac death treating non-cardiac death as competing risk **(F)** for corrected serum calcium. HF, heart failure; Q, quartile.

In competing risk regression analysis, there was a statistically significant trend of rising post-discharge HF risk across quartiles of phosphorus (all *p*-trend < 0.001) (Table 3). Patients in Q4 of phosphorus were 1.77 (95% CI: 1.40–2.22) times more likely to develop post-discharge HF than patients in Q2 before adjustment. After full adjustments, the results of the primary analysis remained robust [sub-distributional hazard ratios (SHR): 1.55, 95% CI: 1.21–1.99] (Table 3 and Figure 4A). Furthermore, stratified analysis showed that there was a consistent prognostic effect of serum phosphorus on post-discharge HF across most subgroups (Table 4).

**Table 3:** Sub-distributional HRs, ORs, or HRs (95% CIs) associated with serum phosphorus for clinical outcomes after acute myocardial infarction.

	**Q1**	**Q2**	**Q3**	**Q4**	***P*** **-trend**
**Phosphorus range (mmol/L)**	** ≤ 1.07**	**1.08–1.21**	**1.21–1.36**	**≥1.36**	
Post-discharge HF^*^					
Unadjusted	0.87 (0.67, 1.13)	1.00 [Reference]	1.11 (0.87, 1.42)	1.77 (1.40, 2.22)	<0.001
Model 1	0.80 (0.62, 1.04)	1.00 [Reference]	1.15 (0.89, 1.47)	1.85 (1.46, 2.35)	<0.001
Model 2	0.81 (0.62, 1.05)	1.00 [Reference]	1.14 (0.89, 1.47)	1.84 (1.45, 2.34)	<0.001
Model 3	0.81 (0.63, 1.06)	1.00 [Reference]	1.16 (0.90, 1.49)	1.85 (1.45, 2.35)	<0.001
Model 4	0.85 (0.65, 1.10)	1.00 [Reference]	1.13 (0.88, 1.46)	1.55 (1.21, 1.99)	<0.001
In-hospital HF^†^, ^**^					
Unadjusted	0.74 (0.59, 0.92)	1.00 [Reference]	1.03 (0.83, 1.27)	2.14 (1.75, 2.61)	<0.001
Model 1	0.65 (0.51, 0.81)	1.00 [Reference]	1.05 (0.84, 1.31)	2.23 (1.80, 2.77)	<0.001
Model 2	0.64 (0.51, 0.81)	1.00 [Reference]	1.04 (0.83, 1.31)	2.23 (1.79, 2.76)	<0.001
Model 3	0.66 (0.52, 0.83)	1.00 [Reference]	1.04 (0.83, 1.31)	2.19 (1.76, 2.72)	<0.001
Model 4	0.66 (0.52, 0.84)	1.00 [Reference]	1.00 (0.79, 1.26)	1.84 (1.47, 2.31)	<0.001
**All-cause death** ^‡^					
Unadjusted	1.24 (0.83, 1.84)	1.00 [Reference]	1.26 (0.85, 1.87)	2.24 (1.57, 3.20)	<0.001
Model 1	1.05 (0.71, 1.56)	1.00 [Reference]	1.34 (0.90, 2.00)	2.47 (1.71, 3.56)	<0.001
Model 2	1.08 (0.73, 1.61)	1.00 [Reference]	1.30 (0.88, 1.94)	2.45 (1.69, 3.53)	<0.001
Model 3	1.06 (0.71, 1.57)	1.00 [Reference]	1.21 (0.82, 1.80)	2.30 (1.59, 3.31)	<0.001
Model 4	1.06 (0.71, 1.58)	1.00 [Reference]	1.09 (0.73, 1.63)	1.59 (1.08, 2.32)	0.027
Cardiac death^*^					
Unadjusted	1.20 (0.72, 1.99)	1.00 [Reference]	1.43 (0.88, 2.32)	2.69 (1.72, 4.19)	<0.001
Model 1	1.02 (0.61, 1.71)	1.00 [Reference]	1.51(0.92, 2.47)	2.94 (1.86, 4.66)	<0.001
Model 2	1.06 (0.63, 1.78)	1.00 [Reference]	1.46 (0.89, 2.38)	2.90 (1.82, 4.61)	<0.001
Model 3	1.04 (0.62, 1.75)	1.00 [Reference]	1.32 (0.80, 2.18)	2.66 (1.67, 4.23)	<0.001
Model 4	1.07 (0.64, 1.81)	1.00 [Reference]	1.18 (0.70, 1.99)	1.68(1.03, 2.75)	0.057

* Values are sub-distributional hazard ratios (95% CIs).

** NT-proBNP in Model 4 was not adjusted for in-hospital HF.

† Values are odd ratios (95% CIs).

‡ Values are hazard ratios (95% CIs).

Unadjusted model adjusted for: none.

Model 1, adjusted for: sex, age, and BMI.

Model 2, further adjusted for: AMI types, smoking status, prior history of hypertension, diabetes, MI, PCI, and CABG.

Model 3, further adjusted for: heart rate, PCI information and multi-vessel disease.

Model 4, further adjusted for: cTNI, NT-proBNP, eGFR, TC, TG, corrected serum calcium, and magnesium.

CI, confidence interval; HF, heart failure; HR, hazard ratio; OR, odd ratio; Q, quartile.

**Table 4:** Stratified analysis of in-hospital HF and post-discharge HF for serum phosphate.

	**In-hospital HF**		**Post-discharge HF**	
	**Odd ratio (95% CI)**	***P* for interaction**	**Sub-distributional hazard ratio (95% CI)**	***P* for interaction**
**Sex**		0.506		0.158
Female	**1.41 (1.23–1.62)**		1.02 (0.88–1.18)	
Male	**1.56 (1.38–1.75)**		**1.25 (1.13–1.39)**	
**Age (years)**		0.681		0.815
<65	**1.38 (1.22–1.57)**		1.13 (0.99–1.28)	
≥65	**1.53 (1.35–1.74)**		1.10 (0.97–1.26)	
**BMI (kg/m** ^**2**^ **)**		0.978		0.906
<25	**1.44 (1.29–1.62)**		1.11 (0.98–1.25)	
≥25	**1.59 (1.38–1.84)**		**1.24 (1.08–1.42)**	
**MI-type**		0.144		0.414
NSTEMI	**1.54 (1.30–1.83)**		1.17 (0.99–1.39)	
STEMI	**1.46 (1.31–1.62)**		**1.12 (1.01–1.25)**	
**Smoking**		0.422		0.412
Never or light smoker	**1.43 (1.24–1.64)**		1.08 (0.94–1.25)	
Current or ex-smoker	**1.57 (1.40–1.77)**		**1.20 (1.07–1.34)**	
**History of hypertension**		0.001		0.606
No	**1.30 (1.14–1.49)**		**1.23 (1.09–1.40)**	
Yes	**1.68 (1.48–1.90)**		1.10 (0.97–1.25)	
**History of diabetes**		0.567		0.378
No	**1.60 (1.43–1.78)**		1.13 (0.99–1.29)	
Yes	**1.31 (1.12–1.53)**		1.10 (0.96–1.27)	
**eGFR(ml/min/1.73m** ^**2**^ **)**		0.028		0.154
<60	**1.72 (1.45–2.02)**		**1.29 (1.12–1.48)**	
≥60	**1.43 (1.28–1.59)**		1.01 (0.89–1.15)	

Bolded values have P-value < 0.05.

BMI, body mass index; CI, confidence interval; eGFR, estimated glomerular filtration rate; HF, heart failure; MI, myocardial infarction; NSTEMI, non-ST-segment elevation myocardial infarction; STEMI, ST-segment elevation myocardial infarction.

#### Serum phosphorus and in-hospital HF

The restricted cubic spline curve showed that serum phosphorus levels had a J-shaped relationship with in-hospital HF (in unadjusted analysis, Figure 3A).

In logistic analysis, compared with patients in Q2, patients in the highest quartile of phosphorus levels had a higher in-hospital HF risk in the unadjusted model (OR: 2.14, 95% CI: 1.75–2.61, *p*-trend < 0.001), and this association was still stable after full adjustments (OR: 1.84, 95% CI: 1.47–2.31, *p*-trend < 0.001; Table 3).

Furthermore, stratified analysis showed that there was a consistent prognostic effect of serum phosphorus on in-hospital HF across most subgroups, which was more pronounced among patients with hypertension (*p* for interaction = 0.001) or eGFR<60 mL/min/1.73 m^2^ (*p* for interaction = 0.028; Table 4).

#### Serum phosphorus and mortality

At the 12-month median follow-up, patients in Q4 of phosphorus levels also had the lowest survival rate (Figure 2B) and the lowest proportion of patients free of cardiac death (Figure 2C). Serum phosphorus levels had a J-shaped relationship with all-cause mortality and cardiac mortality (in unadjusted analysis, Figures 3C,D).

Compared with Q2, the highest quartile of phosphorus had more than a 2-fold increase in all-cause death risk (HR: 2.24, 95% CI: 1.57–3.20; *p-*trend< 0.001) and cardiac death risk (SHR: 2.69, 95% CI: 1.72–4.19; *p-*trend < 0.001) before adjustment, and this association was still stable in all-cause death risk (HR: 1.59, 95% CI: 1.08–2.32, *p*-trend = 0.027) (Table 3 and Figure 4B) and cardiac death risk (SHR: 1.68, 95% CI: 1.03–2.57) after full adjustments, but *p*-trend value was insignificant in cardiac death risk (Table 3 and Figure 4C).

Furthermore, stratified analysis showed that there was a consistent prognostic effect of serum phosphorus on all-cause death or cardiac death across most subgroups, which was more pronounced among non-smokers or patients with eGFR < 60 mL/min/1.73 m^2^ (all *p* for interaction < 0.05; Table 5).

**Table 5:** Stratified analysis of all-cause death and cardiac death for serum phosphate.

	**All-cause death**		**Cardiac death**	
	**Hazard ratio (95% CI)**	***P*** **for interaction**	**Sub-distributional hazard ratio (95% CI)**	***P*** **for interaction**
**Sex**		0.262		0.297
Female	**1.20 (1.02–1.40)**		1.19 (0.97–1.47)	
Male	**1.45 (1.30–1.62)**		**1.52 (1.34–1.73)**	
**Age (years)**		0.984		0.961
<65	**1.29 (1.06–1.57)**		1.31 (0.93–1.85)	
≥65	**1.25 (1.12–1.40)**		**1.31 (1.13–1.51)**	
**BMI (kg/m** ^**2**^ **)**		0.065		0.162
<25	**1.39 (1.25–1.55)**		**1.47 (1.29–1.68)**	
≥25	**1.28 (1.06–1.55)**		1.17 (0.81–1.69)	
**MI-type**		0.442		0.739
NSTEMI	1.16 (0.92–1.47)		1.28 (0.94–1.75)	
STEMI	**1.39 (1.26–1.53)**		**1.40 (1.24–1.58)**	
**Smoking**		0.046		0.048
Never or light smoker	**1.38 (1.20–1.59)**		**1.42 (1.18–1.71)**	
Current or ex–smoker	**1.31 (1.14–1.51)**		**1.36 (1.13–1.65)**	
**History of hypertension**		0.289		0.379
No	**1.37 (1.20–1.57)**		**1.38 (1.18–1.61)**	
Yes	**1.31 (1.15–1.50)**		**1.35 (1.13–1.60)**	
**History of diabetes**		0.252		0.859
No	**1.22 (1.08–1.38)**		**1.25 (1.06–1.47)**	
Yes	**1.68 (1.43–1.98)**		**1.67 (1.37–2.04)**	
**eGFR (ml/min/1.73m** ^**2**^ **)**		0.001		0.046
≥60	**1.48 (1.33–1.65)**		**1.51 (1.33–1.70)**	
<60	1.05 (0.86–1.29)		1.00 (0.69–1.46)	

Bolded values have P-value < 0.05.

BMI, body mass index; CI, confidence interval; eGFR, estimated glomerular filtration rate; HF, heart failure; MI, myocardial infarction; NSTEMI, non-ST-segment elevation myocardial infarction; STEMI, ST-segment elevation myocardial infarction.

#### Serum calcium and post-discharge HF

With increasing quartiles of corrected serum calcium levels, the proportion of patients free of post-discharge HF decreased at the 12-month median follow-up (Figure 2D). The restricted cubic spline curve showed that corrected serum calcium levels had a S-shaped relationship with post-discharge HF (in unadjusted analysis, Figure 3F).

However, in competing risk regression analysis, the association between corrected serum calcium levels and the risk of post-discharge HF was non-significant before adjustments and after full adjustments (Table 6 and Figure 4D). Stratified analysis showed higher corrected serum calcium was related to a lower risk of post-discharge HF in patients ≥65 years old (*p* for interaction = 0.004) (Table 7).

**Table 6:** Sub-distributional HRs, ORs, or HRs (95% CIs) associated with corrected calcium for clinical outcomes after acute myocardial infarction.

	**Q1**	**Q2**	**Q3**	**Q4**	***P*** **-trend**
**Corrected calcium range (mmol/L)**	**≤ 2.09**	**2.09–2.17**	**2.17–2.27**	**≥2.27**	
Post-discharge HF^*^					
Unadjusted	0.91 (0.72, 1.16)	1.00 [Reference]	1.02 (0.81, 1.29)	1.15 (0.91, 1.45)	0.066
Model 1	0.95 (0.75, 1.21)	1.00 [Reference]	0.96 (0.76, 1.22)	0.98 (0.77, 1.24)	0.938
Model 2	0.94 (0.73, 1.19)	1.00 [Reference]	0.96 (0.76, 1.22)	0.98 (0.77, 1.24)	0.810
Model 3	0.93 (0.73, 1.19)	1.00 [Reference]	0.97 (0.77, 1.23)	0.99 (0.78, 1.26)	0.728
Model 4	0.94 (0.73, 1.20)	1.00 [Reference]	0.90 (0.71, 1.15)	0.87 (0.67, 1.11)	0.407
In-hospital HF^†^^**^					
Unadjusted	1.05 (0.84, 1.31)	1.00 [Reference]	1.36 (1.10, 1.68)	2.29 (1.87, 2.82)	<0.001
Model 1	1.14 (0.90, 1.43)	1.00 [Reference]	1.25 (1.00, 1.56)	1.84 (1.49, 2.28)	<0.001
Model 2	1.12 (0.89, 1.41)	1.00 [Reference]	1.25 (1.00, 1.56)	1.84 (1.48, 2.28)	<0.001
Model 3	1.13 (0.90, 1.43)	1.00 [Reference]	1.27 (1.02, 1.59)	1.88 (1.51, 2.33)	<0.001
Model 4	1.10 (0.87, 1.41)	1.00 [Reference]	1.16 (0.92, 1.46)	1.62 (1.29, 2.04)	<0.001
**All-cause death** ^‡^					
Unadjusted	1.00 (0.65, 1.55)	1.00 [Reference]	1.49 (1.00, 2.23)	3.13 (2.18, 4.49)	<0.001
Model 1	1.08 (0.70, 1.67)	1.00 [Reference]	1.39 (0.93, 2.09)	2.51 (1.74, 3.61)	<0.001
Model 2	1.05 (0.68, 1.63)	1.00 [Reference]	1.37 (0.92, 2.06)	2.44 (1.69, 3.51)	<0.001
Model 3	1.08 (0.70, 1.67)	1.00 [Reference]	1.38 (0.92, 2.07)	2.40 (1.66, 3.46)	<0.001
Model 4	1.12 (0.72, 1.74)	1.00 [Reference]	1.29 (0.86, 1.94)	1.99 (1.37, 2.88)	<0.001
Cardiac death^*^					
Unadjusted	1.15 (0.68, 1.94)	1.00 [Reference]	1.37 (0.83, 2.28)	3.25 (2.09, 5.06)	<0.001
Model 1	1.25 (0.74, 2.11)	1.00 [Reference]	1.27 (0.76, 2.11)	2.54 (1.62, 3.97)	<0.001
Model 2	1.22 (0.72, 2.07)	1.00 [Reference]	1.25 (0.75, 2.07)	2.42 (1.54, 3.80)	0.001
Model 3	1.25 (0.73, 2.12)	1.00 [Reference]	1.24 (0.75, 2.07)	2.35 (1.50, 3.71)	0.001
Model 4	1.32 (0.78, 2.24)	1.00 [Reference]	1.15 (0.69, 1.93)	1.87 (1.19, 2.96)	0.047

* Values are sub-distributional hazard ratios (95% CIs).

** NT-proBNP in Model 4 was not adjusted for in-hospital HF.

† Values are odd ratios (95% CIs).

‡ Values are hazard ratios (95% CIs).

Unadjusted model adjusted for: none.

Model 1, adjusted for: sex, age, and BMI.

Model 2, further adjusted for: AMI types, smoking status, prior history of hypertension, diabetes, MI, PCI, and CABG.

Model 3, further adjusted for: heart rate, PCI information and multi-vessel disease.

Model 4, further adjusted for: cTNI, NT-proBNP, eGFR, TC, TG, serum phosphorus, and magnesium.

CI, confidence interval; HF, heart failure; HR, hazard ratio; OR, odd ratio; Q, quartile.

**Table 7:** Stratified analysis of in-hospital HF and post-discharge HF for corrected calcium.

	**In-hospital HF**		**Post-discharge HF**	
	**Odd ratio (95% CI)**	***P*** **for interaction**	**Sub-distributional hazard ratio (95% CI)**	***P*** **for interaction**
**Sex**		0.445		0.160
Female	**1.18 (1.03–1.34)**		0.88 (0.75–1.02)	
Male	**1.17 (1.05–1.30)**		0.98 (0.87–1.10)	
**Age (years)**		0.046		0.004
<65	**1.22 (1.08–1.37)**		1.08 (0.94–1.24)	
≥65	**1.15 (1.02–1.29)**		**0.83 (0.73–0.94)**	
**BMI (kg/m** ^**2**^ **)**		0.621		0.502
<25	**1.16 (1.04–1.29)**		0.96 (0.85–1.08)	
≥25	**1.19 (1.05–1.36)**		0.91 (0.78–1.06)	
**MI-type**		0.787		0.963
NSTEMI	**1.17 (1.01–1.36)**		0.90 (0.76–1.06)	
STEMI	**1.17 (1.06–1.29)**		0.96 (0.86–1.07)	
**Smoking**		0.616		0.992
Never or light smoker	**1.22 (1.07–1.39)**		0.93 (0.80–1.07)	
Current or ex–smoker	**1.16 (1.04–1.29)**		0.94 (0.84–1.06)	
**History of hypertension**		0.880		0.970
No	**1.16 (1.02–1.31)**		0.96 (0.83–1.11)	
Yes	**1.18 (1.05–1.32)**		0.91 (0.81–1.03)	
**History of diabetes**		0.923		0.226
No	**1.15 (1.04–1.27)**		0.97 (0.87–1.09)	
Yes	**1.21 (1.04–1.41)**		0.86 (0.73–1.01)	
**eGFR (ml/min/1.73m** ^**2**^ **)**		0.874		0.337
≥60	**1.36 (1.16–1.60)**		0.91 (0.78–1.06)	
<60	**1.16 (1.06–1.28)**		0.99 (0.88–1.12)	

Bolded values have P-value < 0.05.

BMI, body mass index; CI, confidence interval; eGFR, estimated glomerular filtration rate; HF, heart failure; MI, myocardial infarction; NSTEMI, non-ST-segment elevation myocardial infarction; STEMI, ST-segment elevation myocardial infarction.

#### Serum calcium and in-hospital HF

Corrected serum calcium levels had a J-shaped relationship with in-hospital HF (in unadjusted analysis, Figure 3E). In logistic analysis, patients with Q4 of corrected serum calcium had more than a 2-fold increased risk of in-hospital HF than those in Q2 (OR: 2.29, 95% CI: 1.87–2.82; *p*-trend < 0.001) before adjustment, and this association was still stable after full adjustments (OR: 1.62, 95% CI: 1.29–2.04; *p*-trend < 0.001; Table 6). Furthermore, stratified analysis showed that there was a consistent prognostic effect of corrected serum calcium on in-hospital HF across all subgroups, which was more pronounced among patients <65 years old (*p* for interaction = 0.046) (Table 7).

#### Serum calcium and mortality

At the 12-month median follow-up, patients in Q4 of corrected serum calcium levels also had the lowest survival rate (Figure 2E) and the lowest proportion of patients free of cardiac death (Figure 2F). Corrected serum calcium levels had a J-shaped relationship with all-cause mortality and cardiac mortality (in unadjusted analysis, Figures 3G,H).

There was a statistically significant trend of rising all-cause death and cardiac death risk across quartiles of corrected serum calcium (all *p*-trend < 0.05; Table 6). Patients with Q4 of corrected serum calcium had a more than 200% increased risk of all-cause death (HR: 3.13, 95% CI: 2.18–4.49; *p*-trend < 0.001) or cardiac death (SHR: 3.25, 95% CI: 2.09–5.06; *p*-trend < 0.001) than those with Q2 before adjustment. This association was still stable in all-cause death risk (HR: 1.99, 95% CI: 1.37–2.88; *p*-trend < 0.001) or cardiac death risk (SHR: 1.87, 95% CI: 1.19–2.96; *p*-trend = 0.047) after adjusting for prognostic biomarkers, PCI information, multi-vessel disease and other clinical characteristics at baseline (Table 6 and Figures 4E,F). Moreover, there was a consistent prognostic effect of corrected serum calcium on all-cause mortality or cardiac mortality across most subgroups, which was more pronounced among patients without diabetes (all *p* for interaction < 0.05; Table 8).

**Table 8:** Stratified analysis of all-cause death and cardiac death for corrected calcium.

	**All-cause death**		**Cardiac death**	
	**Hazard ratio (95% CI)**	***P*** **for interaction**	**Sub-distributional hazard ratio (95% CI)**	***P*** **for interaction**
**Sex**		0.674		0.987
Female	**1.28 (1.04–1.57)**		1.30 (0.99–1.70)	
Male	**1.25 (1.07–1.47)**		1.12 (0.90–1.40)	
**Age (years)**		0.054		0.073
<65	**1.51 (1.22–1.87)**		**1.52 (1.11–2.09)**	
≥65	1.16 (0.99–1.34)		1.06 (0.87–1.30)	
**BMI (kg/m** ^**2**^ **)**		0.185		0.860
<25	1.12 (0.95–1.32)		1.15 (0.92–1.43)	
≥25	**1.54 (1.26–1.87)**		1.33 (0.99–1.79)	
**MI–type**		0.236		0.176
NSTEMI	1.07 (0.85–1.34)		0.99 (0.74–1.33)	
STEMI	**1.36 (1.17–1.59)**		**1.34 (1.09–1.65)**	
**Smoking**		0.808		0.563
Never or light smoker	**1.24 (1.03–1.49)**		**1.09 (0.86–1.39)**	
Current or ex–smoker	**1.32 (1.11–1.57)**		**1.35 (1.05–1.74)**	
**History of hypertension**		0.823		0.731
No	**1.25 (1.03–1.51)**		1.15 (0.88–1.50)	
Yes	**1.25 (1.05–1.48)**		**1.17 (0.92–1.49)**	
**History of diabetes**		0.047		0.002
No	**1.37 (1.17–1.60)**		**1.48 (1.20–1.83)**	
Yes	1.07 (0.87–1.33**)**		0.83 (0.61–1.14)	
**eGFR (ml/min/1.73 m** ^**2**^ **)**		0.073		0.094
≥60	**1.20 (1.01–1.44)**		1.12 (0.90–1.39)	
<60	**1.42 (1.18–1.70)**		**1.43 (1.06–1.93)**	

Bolded values have P-value < 0.05.

BMI, body mass index; CI, confidence interval; eGFR, estimated glomerular filtration rate; HF, heart failure; MI, myocardial infarction; NSTEMI, non-ST-segment elevation myocardial infarction; STEMI, ST-segment elevation myocardial infarction.

## Discussion

To the best of our knowledge, the current study is the first to explore the prognostic value of serum phosphorus and calcium on admission in the largest AMI population. The key findings are as follows: (1) Serum phosphorus and calcium levels were associated with biomarkers of prognosis in AMI patients; (2) Higher serum phosphorus levels were independently related to the increased risk of in-hospital HF, post-discharge HF, all-cause death and cardiac death; (3) Higher corrected calcium levels were independently related to a greater risk of in-hospital HF, all-cause death and cardiac death. Our findings provide novel insights into the risk factors for incident HF and poor prognosis in AMI patients.

### Serum phosphorus and HF

Some studies found an independent relationship of higher serum phosphate levels with incident HF risk in patients with previous MI (6, 7), which was similar to our findings. However, few of them focused on the relationship between serum phosphate levels and in-hospital HF, and few of them investigated the effect of serum phosphorus on post-discharge HF by subgroup analysis. Moreover, these studies only made minimal adjustments, and few of them evaluated the correlation of serum phosphorus and prognostic biomarkers.

In the present study, we first demonstrated that higher serum phosphorus levels were independently related to an increased risk of in-hospital HF. The factors leading to in-hospital HF include myocardial damage caused by myocardial necrosis, myocardial stunning and mechanical complications (20). Reactive oxygen species, the inflammatory response, and comorbidities such as chronic kidney disease (CKD) also result in the development of in-hospital HF (20). The occurrence of post-discharge HF is the result of myocardial cell death and scar formation, which leads to chronic neurohumoral activation and ventricular remodeling (20). In our study, we found that serum phosphorus was positively correlated with NT-proBNP, cTNI and hs-CRP and negatively correlated with EF and eGFR, which indicated that serum phosphorus was related to neurohumoral activation, ventricular remodeling, myocardial injury, inflammation, and impaired kidney function after AMI.

Several mechanisms may explain the relationship between elevated serum phosphorus and the increased risk of HF.

First, serum phosphorus is closely related to calcification of blood vessels and valves (21) and arterial stiffness (22). The decrease in vascular compliance caused by serum phosphorus may lead to left ventricular (LV) overload and remodeling, which is the structural basis of HF. In our study, patients with higher phosphorus levels had a higher prevalence of multi-vessel diseases. It has been reported that the risk of ventricular remodeling in patients with multi-vessel diseases is 1.2 times higher than that in patients with single-vessel disease (23). Importantly, in the current study, the correlation between serum phosphorus and HF risk remained robust after adjustment for the prevalence of multi-vessel diseases, which indicates that non-ischemic mechanisms may also contribute to the correlation. In addition, we found that there was a greater prognostic effect of serum phosphorus on in-hospital HF among patients with hypertension, which may be related to arterial stiffness due to higher phosphorus (22). What's more, higher phosphorus was more closely related to in-hospital HF risk in patients with impaired renal function than those with preserved renal function in our study, which was similar to the previous study.

Second, serum phosphorus may have a direct impact on the heart. In animal and human studies, serum phosphorus is independently related to LV mass (24, 25). Higher serum phosphorus levels are associated with greater LV mass in men with stable CVD (25). We also found that elevated serum phosphorus was more closely related to the risk of in-hospital HF in men than women, but there was no interaction in the subgroup analysis. Phosphorus binder therapy improves aortic stiffness and diastolic dysfunction and protects against LV hypertrophy (LVH) (24). The increase in serum phosphorus was related to the decrease in LV diastolic function and LVH in participants without prior HF (26). However, there were non-significant differences in echocardiographic characteristics except EF among the different serum phosphorus quartile groups. The reason for the inconsistent results may be due to different races and populations.

Third, higher serum phosphorus may indirectly influence the heart through its interactions with vitamin D deficiency, high parathyroid hormone (PTH) and fibroblast growth factor 23 (27). Unfortunately, the mechanisms of the above hormones were not evaluated in our study.

### Serum phosphorus and mortality

In the present study, higher serum phosphorus was independently related to a greater risk of all-cause mortality or cardiac mortality, which was similar to prior studies. Importantly, prior studies have shown that higher serum phosphorus is related to an increase in all-cause mortality in MI survivors (8), in STEMI populations (6, 9), or in AMI populations with a small sample size (7), and the relation of higher serum phosphorus and cardiac mortality is showed in CKD (28), which are different from our study. The underlying mechanisms may include vascular calcification, endothelial dysfunction, ventricular hypertrophy, and atherosclerosis (6, 9). What's more, the relationship between elevated serum phosphorus and mortality was more pronounced in patients with impaired kidney function, which suggested impaired kidney function was an important factor for the relationship. It was similar to a prior study, in which higher serum phosphorus is independently associated with the risk of adverse events in patients with AMI, and the association is more pronounced in patients with CKD (7).

### Serum calcium and HF

In our study, we first found that there was an independent association between higher corrected serum calcium levels and the increased risk of in-hospital HF in AMI population. In prior studies, higher serum calcium was independently related to HF risk in a community-based cohort study (11), and elevated serum calcium levels were an independent risk factor for HFpEF in patients with type 2 diabetes mellitus (T2DM) (29). Some potential mechanisms may contribute to the association. First, elevated serum calcium may promote left ventricular diastolic function through the link with blood pressure and various metabolic abnormalities, such as diabetes, obesity, metabolic syndrome (30). Second, prior studies have shown that elevated serum calcium is closely related to left ventricular hypertrophy (31) and vascular stiffness (30). What's more, we found that there were interactions between age and corrected serum calcium for the relationship of corrected serum calcium and HF, which suggested age was an important factor for the relationship.

### Serum calcium and mortality

In the current study, higher serum calcium was independently related to all-cause mortality and -hospital HF and post-discharge HF for serum phosphate. cardiac death after full adjustments in AMI patients without calcium and vitamin D supplements. The possible mechanisms by which elevated serum calcium affects vascular calcification, blood coagulation, altered gene expression induced by effects on arterial wall calcium-sensing receptor (32), and cardiovascular risk factors (30), such as lipid and glucose metabolism. However, Lu et al. (10) found that lower calcium levels are independently related to mortality during hospitalization in STEMI patients. Both the increase and decrease in serum calcium levels are correlated with the increased risk of in-hospital mortality in AMI patients (14). Although our findings were different from these prior studies, our findings were observed in a larger prospective cohort study with a longer follow-up and more confounders were adjusted.

Furthermore, we first investigated the association between serum calcium levels and mortality in different subgroups of traditional cardiovascular risk factors. In the subgroup analysis, there was a greater prognostic effect of increased serum calcium on mortality among patients without diabetes. It is suggested that more attention should be given to the effect of serum calcium levels on the prognosis of AMI patients without diabetes.

## Limitations

There are several limitations of the study. First, this was a single-center study. Most of our research participants come from Northeast China, which limits the universality of our results. Second, at baseline, there were no data about the levels of serum PTH, vitamin D, fibroblast growth factor 23, or intake of calcium and phosphorus in the diet, which were available to clarify the potential mechanism of the observed relationships. Third, we did not repeat the measurement and observe the changes in serum calcium and phosphorus levels, but the baseline phosphorus and calcium levels also provide strong prognostic value in AMI patients. Fourth, confounding may still be caused by residual confounding and unmeasured confounders in these observational data despite the efforts of adjustments.

## Conclusion

Our study demonstrated that serum phosphorus and calcium levels were associated with AMI prognostic biomarkers, higher serum phosphorus was an independent predictor of a higher incidence of in-hospital HF, post-discharge HF, all-cause mortality and cardiac mortality, and higher serum calcium was independently related to the increased risk of in-hospital HF, all-cause mortality, and cardiac mortality in AMI patients. These findings will be beneficial to the early risk stratification of incident HF and death after AMI and early standardized treatment of post-AMI HF.

## Data availability statement

The original contributions presented in the study are included in the article/Supplementary material, further inquiries can be directed to the corresponding authors.

## Ethics statement

The studies involving human participants were reviewed and approved by Ethics Committee of Harbin Medical University (Reference Number: KY2017-249). The patients/participants provided their written informed consent to participate in this study.

## Author contributions

WC designed the analysis and wrote the original draft. YL and XZ conducted the model parameterization and the statistical analyses. YW, BZ, YanZ, and XL collected the data. YaoZ and BY provided resources and supervised the study. YaoZ, BY, and SF revised the manuscript. All authors have read and approved the final manuscript.

## Funding

This research was supported by the National Key Research and Development Program of China to BY, grant number 2016YFC1301100; the National Natural Science Foundation of China to YaoZ, grant number 81770255; the National Key Research and Development Program of China to YaoZ, grant numbers 2016YFC1301001, 2016YFC1301002, and 2016YFC1301004; and the Key Laboratory of Myocardial Ischemia, Harbin Medical University of Education to YaoZ, grant number KF202103.

## Conflict of interest

The authors declare that the research was conducted in the absence of any commercial or financial relationships that could be construed as a potential conflict of interest.

## Publisher's note

All claims expressed in this article are solely those of the authors and do not necessarily represent those of their affiliated organizations, or those of the publisher, the editors and the reviewers. Any product that may be evaluated in this article, or claim that may be made by its manufacturer, is not guaranteed or endorsed by the publisher.
