# Corrigendum: Higher serum phosphorus and calcium levels provide prognostic value in patients with acute myocardial infarction

**DOI:** 10.3389/fcvm.2022.1039864

**Published:** 2022-09-30

**Authors:** Wei Cao, Yilan Li, Yao Wen, Shaohong Fang, Bing Zhao, Xiaoyuan Zhang, Yanxiu Zhang, Xueyan Lang, Bo Yu, Yao Zhang

**Affiliations:** ^1^Department of Cardiology, Second Affiliated Hospital of Harbin Medical University, Harbin, China; ^2^Department of Cardiology, Heilongjiang Provincial Hospital, Harbin, China; ^3^Myocardial Ischemia, Ministry of Education, Harbin Medical University, Harbin, China

**Keywords:** serum phosphate, serum calcium, acute myocardial infarction, prognosis, heart failure, mortality

In the published article, there was an error in correspondence email. Instead of “ dryu_hmu@163.com,” it should be “ yubodr@163.com.”

In the published article, there was an error in Table 4 as published. The value of “*P* for interaction” in the subgroup “Sex” was given as “ <0.506.” The correct value is “0.506”. The corrected Table 4 and its caption appear below.

**Table 4:** Stratified analysis of in-hospital HF and post-discharge HF for serum phosphate.

	**In-hospital HF**		**Post-discharge HF**	
	**Odd ratio (95% CI)**	***P* for interaction**	**Sub-distributional hazard ratio (95% CI)**	***P* for interaction**
**Sex**		0.506		0.158
Female	**1.41 (1.23–1.62)**		1.02 (0.88–1.18)	
Male	**1.56 (1.38–1.75)**		**1.25 (1.13–1.39)**	
**Age (years)**		0.681		0.815
<65	**1.38 (1.22–1.57)**		1.13 (0.99–1.28)	
≥65	**1.53 (1.35–1.74)**		1.10 (0.97–1.26)	
**BMI (kg/m** ^**2**^ **)**		0.978		0.906
<25	**1.44 (1.29–1.62)**		1.11 (0.98–1.25)	
≥25	**1.59 (1.38–1.84)**		**1.24 (1.08–1.42)**	
**MI-type**		0.144		0.414
NSTEMI	**1.54 (1.30–1.83)**		1.17 (0.99–1.39)	
STEMI	**1.46 (1.31–1.62)**		**1.12 (1.01–1.25)**	
**Smoking**		0.422		0.412
Never or light smoker	**1.43 (1.24–1.64)**		1.08 (0.94–1.25)	
Current or ex-smoker	**1.57 (1.40–1.77)**		**1.20 (1.07–1.34)**	
**History of hypertension**		0.001		0.606
No	**1.30 (1.14–1.49)**		**1.23 (1.09–1.40)**	
Yes	**1.68 (1.48–1.90)**		1.10 (0.97–1.25)	
**History of diabetes**		0.567		0.378
No	**1.60 (1.43–1.78)**		1.13 (0.99–1.29)	
Yes	**1.31 (1.12–1.53)**		1.10 (0.96–1.27)	
**eGFR(ml/min/1.73m** ^**2**^ **)**		0.028		0.154
<60	**1.72 (1.45–2.02)**		**1.29 (1.12–1.48)**	
≥60	**1.43 (1.28–1.59)**		1.01 (0.89–1.15)	

Bolded values have P-value < 0.05.

BMI, body mass index; CI, confidence interval; eGFR, estimated glomerular filtration rate; HF, heart failure; MI, myocardial infarction; NSTEMI, non-ST-segment elevation myocardial infarction; STEMI, ST-segment elevation myocardial infarction.

In the published article, there were two errors. The statement for the method of statistics analysis was given as “Kruskal–Wallis,” where it should be “Kruskal-Wallis test.”

A correction has been made to Methods, **Statistics analysis**. This sentence previously stated:

“…compared by Kruskal–Wallis chi-square test or Fisher's exact test.”

The corrected sentence appears below:

“…compare by Kruskal–Wallis test, chi-square test or Fisher's exact test.”

A correction has been made to Discussion, **Serum phosphorus and mortality**. This sentence previously stated:

“It was similar to a prior study, in which higher serum phosphorus is independently associated with the risk of adverse events in patients with AMI, and the association is more pronounced in patients with CKD (7). This phenomenon may be attributed to the reduced sample size when the patients were divided into different subgroups, or due to different races, baseline characteristics, and controlling for more confounders.”

The corrected sentence appears below:

“It was similar to a prior study, in which higher serum phosphorus is independently associated with the risk of adverse events in patients with AMI, and the association is more pronounced in patients with CKD (7).”

The authors apologize for these errors and state that this does not change the scientific conclusions of the article in any way. The original article has been updated.

## Publisher's note

All claims expressed in this article are solely those of the authors and do not necessarily represent those of their affiliated organizations, or those of the publisher, the editors and the reviewers. Any product that may be evaluated in this article, or claim that may be made by its manufacturer, is not guaranteed or endorsed by the publisher.

