# Supplementary material for: Higher serum phosphorus and calcium levels provide prognostic value in patients with acute myocardial infarction
Source: Front Cardiovasc Med. 2022 Sep 7;9:929634. doi: 10.3389/fcvm.2022.929634 (PMC9489914; doi:10.3389/fcvm.2022.929634)
Supplement: Supplementary file 1 [file Table_1.DOCX]

Supplementary Material

# Supplementary Figures and Tables

## Supplementary Tables

**Supplemental Table 1.** Correlation between variables and phosphorus, corrected calcium in patients with acute myocardial infarction

|  | Serum phosphorus | | Corrected calcium | |
| --- | --- | --- | --- | --- |
|  | Correlation coefficient (r ) | P value | Correlation coefficient (r ) | P value |
| cTNI | 0.058 | < 0.001 | -0.110 | < 0.001 |
| NT-ProBNP | 0.171 | < 0.001 | 0.230 | < 0.001 |
| hs-CRP | 0.066 | < 0.001 | 0.207 | < 0.001 |
| eGFR | -0.044 | 0.006 | -0.225 | < 0.001 |
| EF | -0.094 | < 0.001 | -0.095 | < 0.001 |
| Corrected calcium | 0.023 | 0.146 | - | - |

cTNI, cardiac troponin I; EF, ejection fraction; eGFR, estimated glomerular filtration rate; hs-CRP, high-sensitivity C reactive protein; NT-proBNP, N-terminal pro-B-type natriuretic peptide.
